# Case Report: A rare case of multi-vessel coronary artery-coronary sinus fistula combined with multiple coronary aneurysms initially presenting with frequent premature ventricular complexes

**DOI:** 10.3389/fcvm.2026.1920885

**Published:** 2026-08-11

**Authors:** Hongwei Yi, Jia Li, Yue Bao

**Affiliations:** Department of Cardiology, Wuhan Asia Heart Hospital, Wuhan, China

**Keywords:** computed tomography angiography, coronary artery aneurysm, coronary artery fistula, coronary sinus, premature ventricular complex

## Abstract

Coronary artery fistula (CAF) is a rare congenital coronary malformation. Sakakibara type B lesions, in which multiple coronary arteries drain concurrently into the coronary sinus (CS), are extremely uncommon, and cases initially presenting with high-burden premature ventricular complexes (PVCs) as the initial manifestation have rarely been reported. This paper reports a 63-year-old female patient presenting with intermittent palpitations for 1 year. A 24-hour ambulatory electrocardiogram (Holter) revealed a PVC burden of up to 40,804 complexes, with a total of 101,582 heartbeats recorded. Transthoracic echocardiography (TTE) demonstrated dilation of the left atrium, left ventricle and right atrium, moderate mitral and tricuspid regurgitation, dilated right coronary artery (RCA) with a right coronary artery-coronary sinus fistula, while lesions of the left circumflex artery (LCX) and second obtuse marginal branch (OM2) were missed. Three-dimensional coronary computed tomography angiography (CCTA) reconstruction showed tortuous and diffusely dilated LCX, OM2 and RCA, with aneurysms at the distal segments of all three vessels. The distal segments of the three vessels anastomosed with each other and drained into the CS. The dilated coronary arteries compressed the great cardiac vein, resulting in focal luminal stenosis. The patient's PVCs originated from the left coronary cusp. Radiofrequency catheter ablation completely relieved palpitations, and follow-up Holter at 3 months recorded only 1 PVC with a different origin from preoperative ectopy. Cardiac surgeons recommended surgical ligation of the fistula, but the patient opted for regular imaging follow-up and long-term clopidogrel antiplatelet therapy due to absence of myocardial ischemia, heart failure and other symptoms. This case indicates that three-dimensional CCTA reconstruction serves as the primary non-invasive modality for definitive diagnosis and therapeutic evaluation of complex CAF. Idiopathic PVCs originating from the aortic sinuses of Valsalva can coexist independently with CAF. Type B CAF with multiple feeding vessels draining into the CS represents a high-risk anatomical subtype, requiring long-term, individualized multidisciplinary management.

## Introduction

Coronary artery fistula (CAF) was first described by Krause in 1865 (1). It refers to an abnormal shunt channel between the main coronary artery/branches and cardiac chambers, pulmonary artery or coronary vein, where blood bypasses myocardial capillary networks and directly flows into low-pressure vessels or cardiac chambers. The population incidence of CAF ranges from 0.002% to 0.3%, with a detection rate of 0.2%–0.3% on coronary angiography. Congenital CAF accounts for 90.7% of cases, with incomplete regression of embryonic myocardial sinusoids as the core pathogenesis; iatrogenic acquired fistulas make up only 8.3%, mainly induced by cardiac valve surgery, coronary artery bypass grafting, myocardial biopsy, thoracic trauma and other procedures (2).

Based on angiographic morphology, Sakakibara et al. classified CAF into two types: Type A (proximal fistula), with dilation limited to the coronary segment proximal to the fistula orifice and normal distal vessel caliber; Type B (distal fistula), with tortuous dilation of the entire coronary artery from its origin to the drainage ostium (3). Approximately 90% of fistulas drain into the right heart circulation, most commonly the right ventricle, followed by the right atrium and pulmonary artery; fistulas draining into the coronary sinus (CS) account for roughly 7%–11%. Around 80.4% of CAF patients have single-vessel lesions, while only 19.6% present with multi-vessel fistulas (2, 4). Type B fistulas involving simultaneous multiple coronary arteries converging into the CS are extremely rare in clinical practice.

Chronic left-to-right shunting persistently elevates shear stress within coronary arteries, damaging vascular wall structures and progressively causing luminal dilation and tortuosity, which may further lead to coronary artery aneurysms in severe cases. Patients with minimal shunt volume may remain asymptomatic for decades, whereas those with large shunts present with exertional palpitations, chest tightness and dyspnea on exertion. Long-term complications include heart failure, coronary steal syndrome, thromboembolism and even aneurysm rupture (2, 4).

Premature ventricular complexes (PVCs) originating from the aortic sinuses of Valsalva are common idiopathic arrhythmias. However, cases featuring high-burden PVCs as the chief complaint combined with multi-vessel coronary artery-coronary sinus fistulas and multiple aneurysms have scarcely been documented. This article integrates complete imaging, electrophysiological, therapeutic and follow-up data of this case, combined with evidence from recent large-scale pooled analyses and single-center cohort studies for in-depth discussion.

## Case presentation

### General information

**Figure 1:**
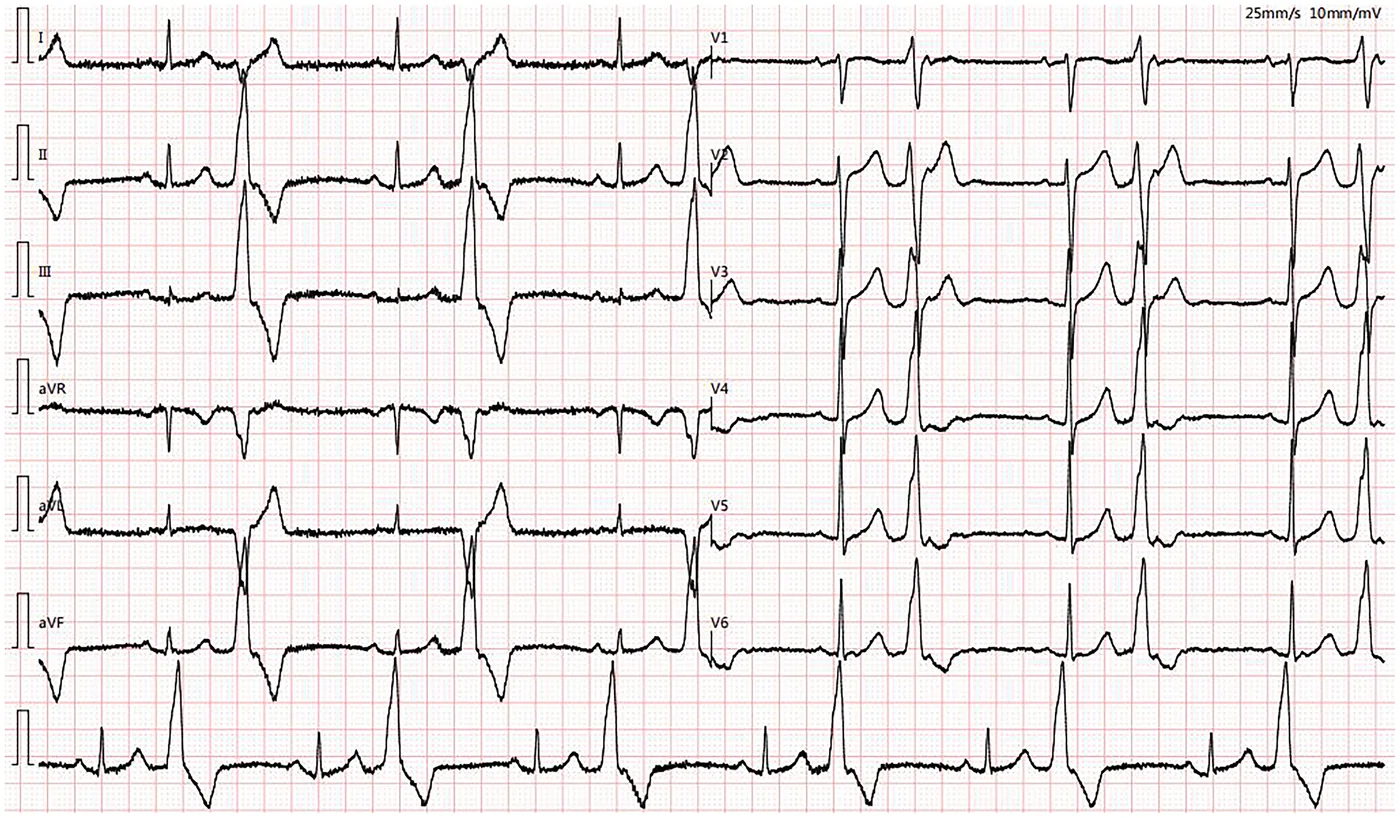
ECG showing sinus rhythm with PVC bigeminy. The QRS morphology demonstrates positive deflections in inferior leads (II, III, aVF) and an earlier precordial transition of PVCs relative to sinus QRS complexes, consistent with PVCs originating within the sinuses of Valsalva.

**Figure 2:**
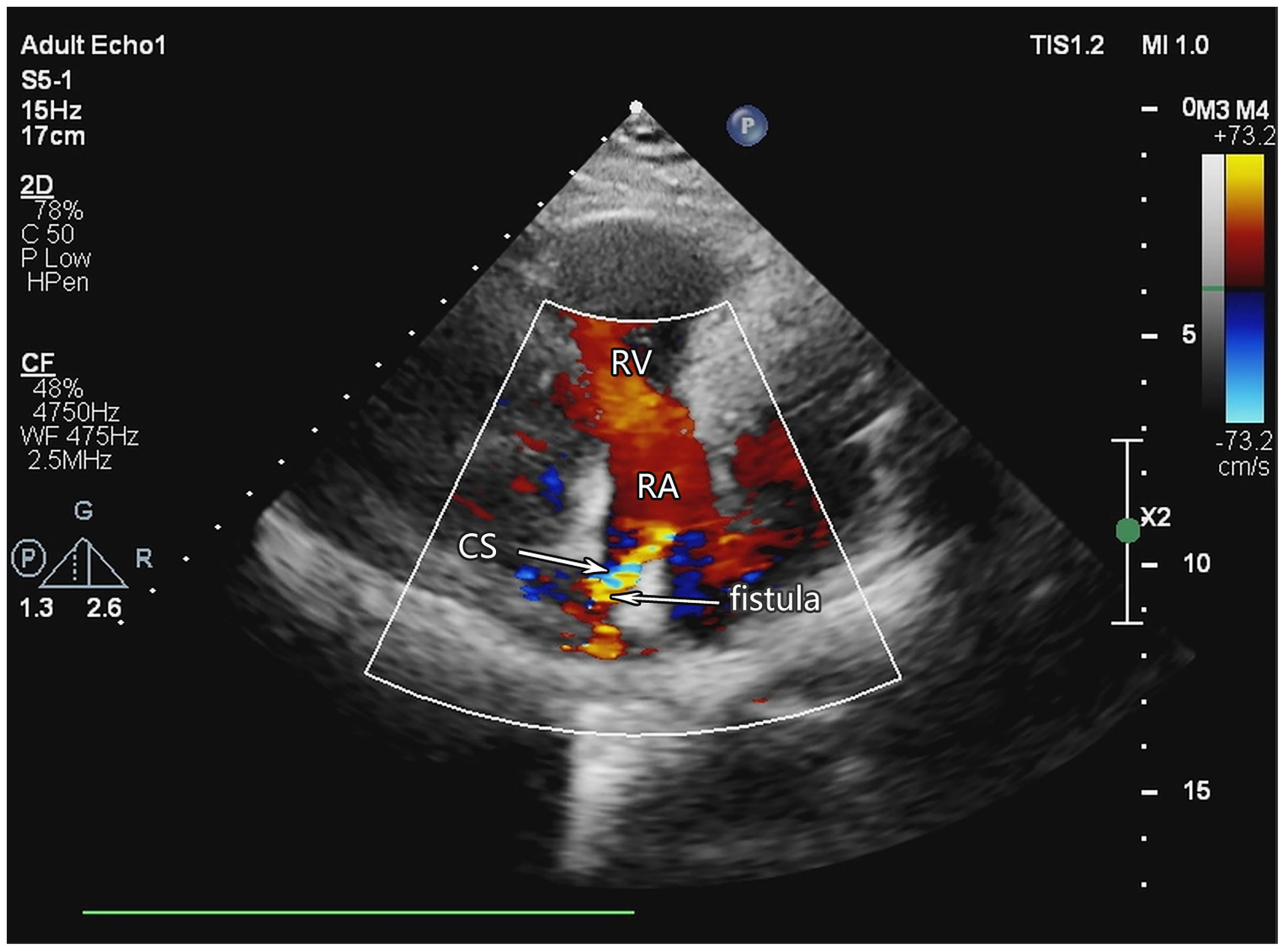
TTE showing coronary artery-coronary sinus fistula. RV, right ventricular; RA, right atrial; CS, coronary sinus.

A 63-year-old female patient presented with a chief complaint of intermittent palpitations lasting 1 year. Palpitations manifested as irregular heart rhythm, aggravated by physical activity and alleviated at rest, severely impairing quality of daily life. Outpatient 12-lead electrocardiogram (ECG) revealed ventricular bigeminy composed of PVCs (Figure 1). A 24-hour Holter recorded a total of 101,582 heartbeats, including 40,804 monomorphic PVCs. Transthoracic echocardiography (TTE) showed: left atrial anteroposterior diameter 42 mm, right atrial diameter 46 mm, left ventricular end-diastolic diameter 54 mm, right ventricular diameter 38 mm, left ventricular ejection fraction 55%, moderate systolic mitral and tricuspid regurgitation. The right coronary artery (RCA) measured 3 mm at its origin and 8 mm at the mid-segment, opening into the wall of the CS with a continuous high-velocity shunt jet entering the CS at a flow velocity of 3.4 m/s and trans-fistula pressure gradient of 48 mmHg. Echocardiographic diagnosis: left cardiac chamber dilation, right atrial dilation, moderate mitral and tricuspid regurgitation, coronary artery-coronary sinus fistula and dilated RCA (Figure 2, Supplementary Videos 1, 2). The patient was admitted for further treatment.

### Admission physical examination

Temperature 36.5 °C, pulse 78 beats/min, respiratory rate 18 breaths/min, blood pressure 130/76 mmHg. The patient was alert without jugular venous distension. Bilateral lung auscultation revealed clear breath sounds without rales or rhonchi. Mild cardiomegaly was noted on percussion, with irregular heart rate at 78 beats/min. No pathognomonic continuous machinery murmur of CAF was heard over any valvular auscultation areas. Abdomen was soft with no hepatosplenomegaly, and no bilateral lower extremity edema. She had no prior history of hypertension, diabetes, thoracic trauma or family history of congenital heart disease. Laboratory tests including complete blood count, liver function, renal function, electrolytes, myocardial enzymes, coagulation profile and thyroid function were all within normal limits.

**Figure 3:**
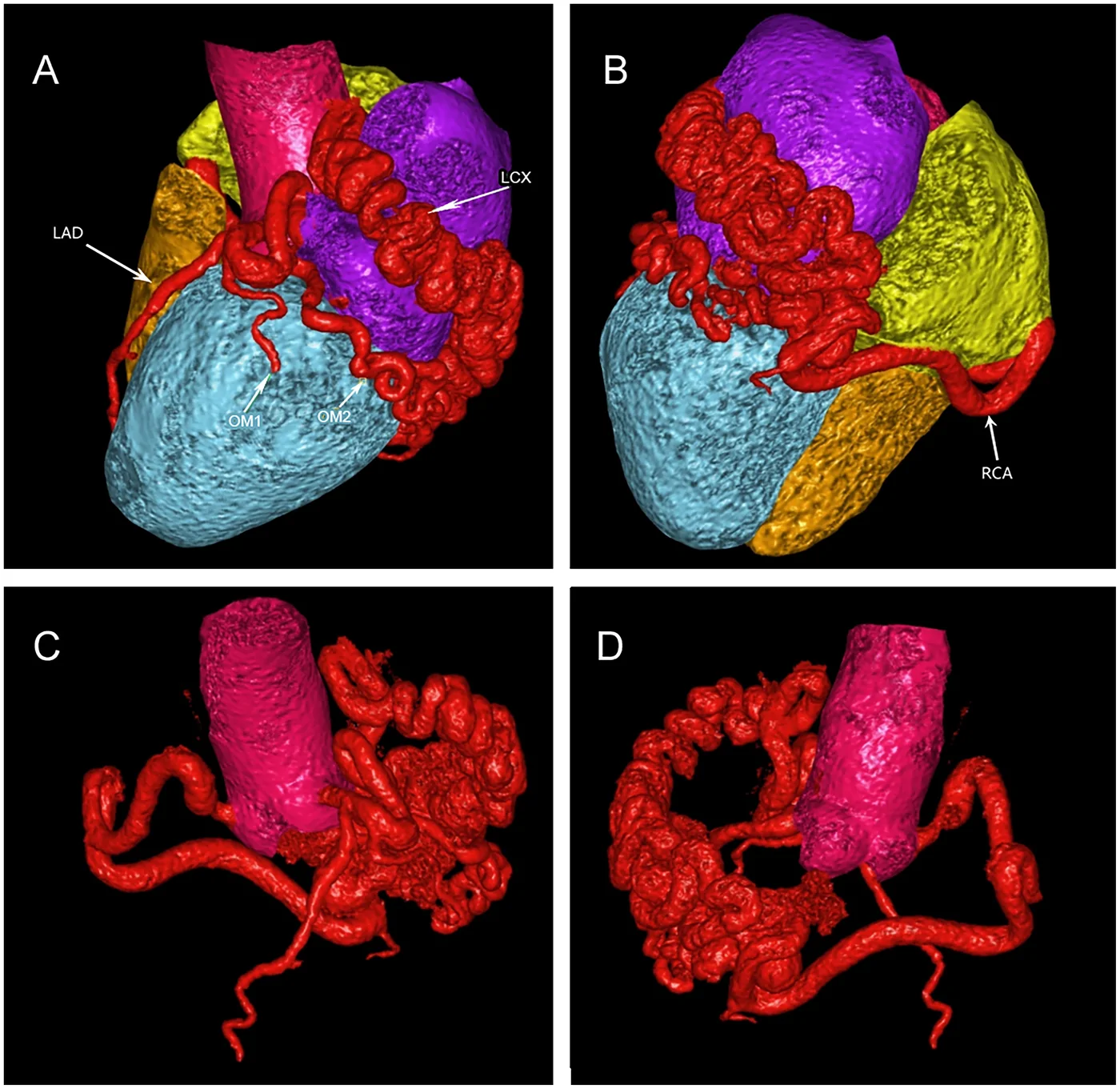
Three-dimensional CCTA reconstruction of coronary arteries and cardiac chambers. **(A,B)**: Whole heart with coronary arteries. **(A)** Anterior cardiac view; **(B)** Posterior cardiac view. **(C,D)** Isolated tortuous and dilated coronary arteries. **(C)** Anterior view; **(D)** Posterior view. LAD, left anterior descending artery; LCX, left circumflex artery; OM1, first obtuse marginal branch; OM2, second obtuse marginal branch; RCA, right coronary artery.

Three-dimensional coronary computed tomography angiography (CCTA) reconstruction demonstrated diffuse tortuosity and significant luminal widening of the left circumflex artery (LCX), with caliber ranging from 6.3 mm to 11.8 mm and a focal distal aneurysm with maximum diameter 12.0 mm. The first obtuse marginal branch (OM1) showed mild proximal dilation, while the second obtuse marginal branch (OM2) was tortuously dilated with a caliber of 5.7–8.2 mm. The entire RCA was dilated (8.7–9.9 mm), with a distal aneurysm measuring up to 14.6 mm in maximum diameter. The distal segments of LCX, OM2 and RCA anastomosed with one another and drained collectively into the CS via a common anastomotic ostium of 9.2 mm. The dilated coronary arteries compressed the great cardiac vein, causing focal luminal stenosis (Figure 3). Combined findings of TTE and CCTA confirmed the diagnosis: tortuous dilation of three coronary arteries (Sakakibara Type B), multiple coronary artery aneurysms, coronary artery-coronary sinus fistula, concomitant partial cardiac chamber dilation and moderate mitral and tricuspid regurgitation.

### Treatment and clinical outcome

Multidisciplinary cardiac surgery consultation recommended surgical ligation of the fistula for this Type B multi-vessel CAF with multiple coronary aneurysms, followed by long-term antiplatelet therapy to reduce intralesional thrombus risk. After full disclosure of surgical benefits, perioperative risks, and pros/cons of conservative surveillance, the patient declined surgery and elected long-term oral antiplatelet therapy with serial imaging follow-up for coronary morphology, cardiac structure, and ventricular function.

Given the extremely high PVC burden and severe palpitations limiting daily activities, the patient met the radiofrequency ablation indications for ventricular arrhythmias outlined in the 2022 European Society of Cardiology (ESC) guidelines (5). ECG QRS morphology localized the ectopy to the aortic sinuses of Valsalva, an anatomical site associated with high procedural success and low complication risk for ablation. On hospital day 3, intracardiac electrophysiology study and radiofrequency catheter ablation were performed under local anesthesia. Non-selective aortic angiography was first conducted to delineate aortic sinuses of Valsalva anatomy and left main coronary ostium, revealing generalized tortuosity of bilateral coronary arteries (Supplementary Videos 3, 4). Combined pace mapping and activation mapping identified the earliest local activation within the left coronary cusp, occurring 25 ms earlier than the surface QRS complex. The target site was approximately 15 mm distal to the left main coronary ostium. All PVCs disappeared after 10 s of radiofrequency delivery at 35 W, followed by four supplementary applications lasting 60–120 s each.

No PVCs were observed on post-procedure cardiac telemetry, and palpitations resolved completely. The patient was discharged after uncomplicated puncture site healing, with lifelong clopidogrel 75 mg once daily prescribed. Three-month outpatient follow-up Holter recorded a total of 98,176 heartbeats with only 1 PVC of distinct morphology from preoperative ectopy, consistent with a separate ectopic origin. Follow-up TTE showed no significant changes in cardiac structure compared with baseline. Annual TTE surveillance was recommended to assess cardiac chamber size and valvular regurgitation, with CCTA repeated every 2 years to monitor aneurysm and fistula morphology; follow-up intervals would be individualized based on disease progression. Surgical re-evaluation would be performed in the event of progressive aneurysm enlargement, declining ventricular function or new-onset myocardial ischemia symptoms.

**Figure 4:**
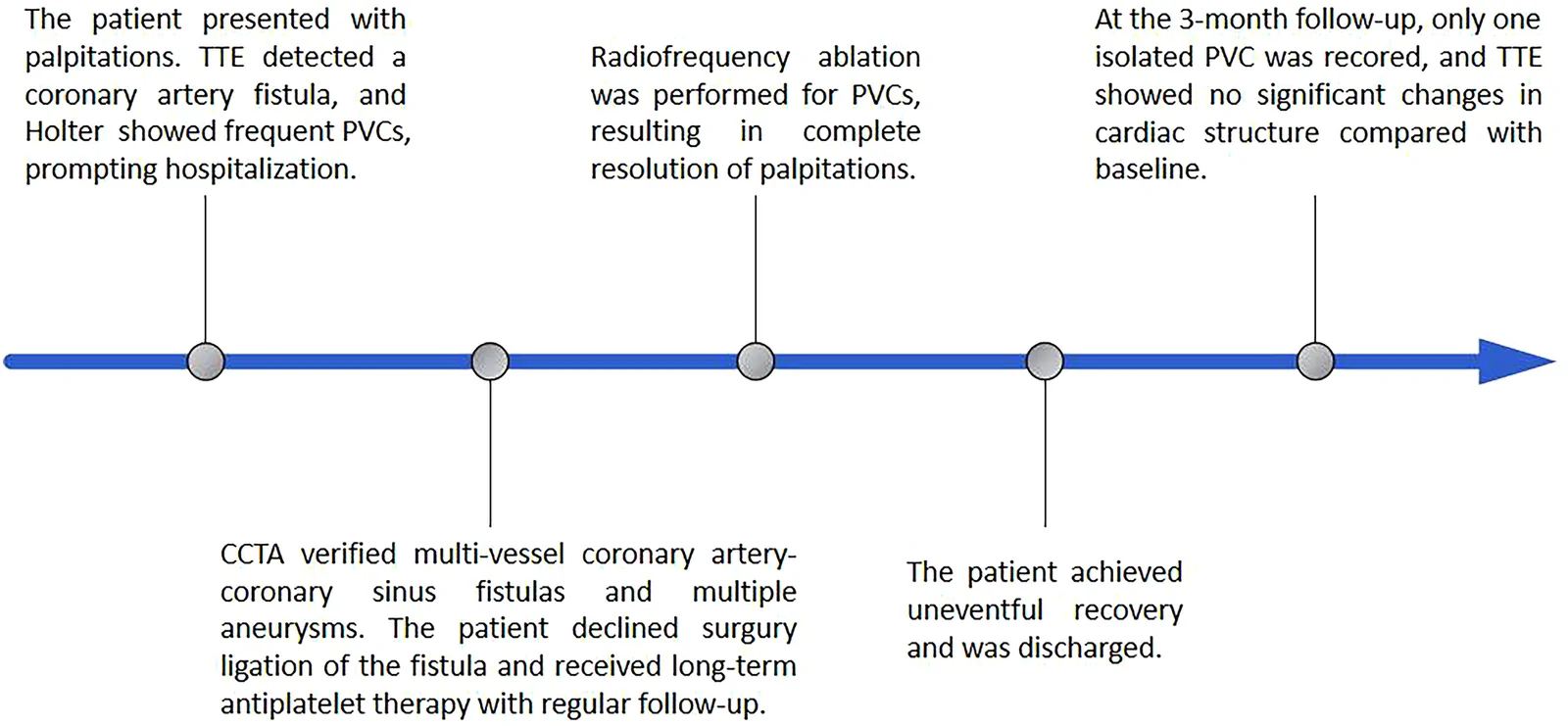
The timeline of the patient's management.

The timeline of the patient’s management is summarized in Figure 4.

## Discussion

This case represents anatomically complex Sakakibara Type B CAF, with three coronary arteries anastomosing distally to form a collective drainage tract into the CS, generating substantial shunt volume that induced diffuse coronary tortuosity and multiple aneurysms. Fistula ostium size and pulmonary-to-systemic blood flow ratio (Qp/Qs) are independent risk factors for secondary coronary vascular injury, with larger shunt volumes conferring higher long-term vascular complication risks (2).

Classic CAF typically presents with a pathognomonic continuous machinery cardiac murmur, which requires superficial fistula course, significant pressure gradient across the shunt and absence of overlying tissue attenuation. In this patient, multi-vessel coronary branches converged distally to drain into the posteriorly located CS via a large ostium; pressure gradients decayed rapidly as shunted blood entered the low-pressure coronary venous system. Furthermore, the tortuous dilated coronary arteries were encased within atrial and ventricular myocardium, preventing turbulent murmurs from transmitting to the precordium, and hence no characteristic murmur was detected on physical examination.

TTE serves as a first-line screening tool for CAF and reliably identifies large-caliber fistulas, yet exhibits poor resolution for complex multi-branch CAF with high missed diagnosis rates (6). Preoperative TTE only detected the RCA-CS fistula while missing LCX and OM2 lesions, confirming this imaging limitation. This finding aligns with a 2024 large-scale angiographic cohort study demonstrating that TTE yields a diagnostic accuracy of merely 9% for multi-branch, complex CAF with high false-negative rates for small, segmental lesions (7). Three-dimensional CCTA reconstruction represents a precise, non-invasive imaging modality capable of stereoscopically delineating coronary branch anatomy, fistula geometry, distal drainage pathways, while accurately measuring aneurysm diameter, vascular stenosis and adjacent compressive effects to facilitate definitive Sakakibara classification; it is the preferred non-invasive pre-procedural evaluation tool for complex multi-vessel CAF. Recent reviews have validated the utility of patient-specific 3D printed models generated from CCTA datasets to assist pre-operative anatomical planning and device selection for complex CAF, lowering intraprocedural risks (8). Invasive coronary angiography enables dynamic assessment of coronary flow reserve and concurrent transcatheter closure, yet two-dimensional projection fails to fully visualize three-dimensional vascular architecture. Therefore, following initial TTE detection of CAF, CCTA is prioritized for anatomical subtyping, with invasive angiography reserved for hemodynamic characterization and interventional planning when indicated.

No unified treatment algorithm exists for CAF, and clinical management requires risk stratification based on patient symptoms, fistula ostium size, shunt volume, vascular morphology and ventricular function. Small CAF lesions in infants carry a 44% risk of spontaneous closure, whereas spontaneous closure occurs in <1% of adult CAF patients (4, 9). Long-term surveillance alone is acceptable for patients with micro-fistulas, no aneurysms, Qp/Qs <1.5, no cardiac chamber dilation and absence of clinical symptoms (2, 4). Multiple clinical reviews confirm that CAF draining into the coronary sinus constitutes a high-risk anatomical subtype associated with markedly elevated long-term risks of thrombosis and myocardial infarction (10). Definitive fistula obliteration is indicated for patients with prominent symptoms, large shunt volumes, giant coronary aneurysms or progressive ventricular dysfunction to mitigate adverse cardiovascular events. Two interventional strategies are available: percutaneous transcatheter closure and open surgical repair. Transcatheter closure is reserved for uncomplicated single-channel fistulas with regular anatomy, utilizing three primary device categories: embolization coils, patent ductus arteriosus (PDA) occluders and vascular plugs. Single-center comparative studies report an acute procedural success rate of 87.9% for PDA occluders in single-ostium lesions with large aneurysms, superior to 62.9% with embolization coils; coils are better suited for multi-origin, highly tortuous micro-fistulous tracts (11). Surgical ligation is indicated for multi-vessel involvement, multiple fistulous ostia, giant aneurysms, diffuse distal Type B dilation, failed transcatheter closure, severely tortuous vasculature or concomitant additional congenital cardiac malformations (2). While the largest aneurysm in this patient measured 14.6 mm, below widely accepted adult criteria for giant coronary aneurysms (12), the complex three-vessel anastomosis forming a broad common drainage ostium rendered complete percutaneous device exclusion of all feeding branches unfeasible, with high residual shunt and recanalization risks, establishing surgical ligation as the optimal curative strategy.

The patient's primary clinical complaint of palpitations was entirely attributable to high-burden PVCs, for which radiofrequency ablation represents a definitive therapeutic intervention. ECG QRS morphology localized ectopy to the aortic valve cusps (left, right or non-coronary cusp), a well-established origin site for idiopathic ventricular arrhythmias driven by enhanced automaticity or micro-reentry within focal myocardial tissue (5). No clinical or imaging evidence of myocardial ischemia or fibrosis was identified in this patient, and anatomical separation between the coronary fistula/aneurysms and the aortic sinuses of Valsalva ablation target eliminated mechanical stimulation via vascular compression or flow shear stress as an underlying trigger. Current evidence supports the coexistence of two independent cardiovascular pathologies without direct pathogenetic linkage.

The large cumulative shunt volume from three coronary arteries draining into the CS induced diffuse coronary tortuosity and multiple aneurysms, serving as a major driver of cardiac chamber dilation and valvular regurgitation. Concurrently, the patient's high PVC burden predisposed to PVC-induced cardiomyopathy, exacerbating ventricular dilation and regurgitant severity (5, 13). We hypothesize that cardiac chamber enlargement and valvular dysfunction arose from combined hemodynamic stress of CAF left-to-right shunting and chronic frequent PVCs. Radiofrequency ablation eliminates PVC burden and interrupts the pathological pathway of PVC-mediated myocardial remodeling, potentially facilitating reverse ventricular remodeling while enabling differentiation of the independent contributions of each pathological process to cardiac dysfunction. While palpitations resolved completely post-ablation, no evidence of reverse ventricular remodeling was observed at 3-month follow-up; serial monitoring of cardiac chamber dimensions and regurgitation severity will continue long-term.

The patient's severe coronary tortuosity, multiple aneurysms and broad fistulous ostium confer elevated thromboembolic risk, rendering surgical fistula ligation the preferred curative intervention with adjuvant lifelong clopidogrel antiplatelet therapy post-operatively. Nevertheless, the patient opted for conservative serial imaging surveillance following comprehensive physician-patient discussion of risks and benefits, based on her current asymptomatic status. This individualized decision reflects patient preference under asymptomatic conditions and does not constitute standard management for this high-risk anatomical subtype of CAF. Longitudinal surveillance of coronary artery morphology and aneurysm diameter will continue, with timely therapeutic adjustment implemented upon disease progression.

This single-case retrospective analysis bears inherent limitations. First, invasive cardiac catheterization was not performed to quantitatively measure Qp/Qs shunt ratio, lacking objective hemodynamic load quantification. Second, the patient elected conservative management with limited follow-up duration, precluding definitive conclusions regarding long-term aneurysm progression, thromboembolic event risk and the efficacy of clopidogrel for thrombosis prophylaxis in multiple coronary aneurysms; findings cannot be generalized to comparable patient cohorts. Third, despite the absence of a direct causal relationship between CAF and ventricular ectopy hypothesized herein, genetic testing was not completed to exclude underlying hereditary coronary developmental anomalies as a shared congenital predisposition.

## Conclusions

Premature ventricular complexes originating from the aortic sinuses of Valsalva are typically idiopathic arrhythmias that may occur in patients without structural heart disease. This patient presented with symptomatic PVCs as the initial manifestation, and incidental imaging identified complex Sakakibara Type B multi-vessel coronary artery-coronary sinus fistula complicated by multiple coronary aneurysms, with the two pathologies likely unrelated in pathogenesis. Radiofrequency catheter ablation targeting ventricular ectopy achieved complete resolution of palpitations. Type B distal CAF with multiple feeding vessels draining into the coronary sinus represents a high-risk anatomical subtype associated with elevated long-term risks of thrombosis, aneurysm rupture and myocardial infarction. Surgical ligation is prioritized for anatomically complex lesions not amenable to complete transcatheter closure. Asymptomatic patients require joint multidisciplinary risk-benefit assessment by cardiologists and cardiac surgeons to establish individualized long-term imaging surveillance protocols, enabling dynamic monitoring of vascular and ventricular function with prompt intervention upon disease progression. The diagnostic and therapeutic workflow of this case provides clinical decision-making reference for analogous rare congenital coronary malformations.

## Data Availability

The raw data supporting the conclusions of this article will be made available by the authors, without undue reservation.
